# Automated Training for Algorithms That Learn from Genomic Data

**DOI:** 10.1155/2015/234236

**Published:** 2015-01-28

**Authors:** Gokcen Cilingir, Shira L. Broschat

**Affiliations:** ^1^School of Electrical Engineering and Computer Science, Washington State University, Pullman, WA 99164, USA; ^2^Department of Veterinary Microbiology and Pathology, Washington State University, Pullman, WA 99164, USA; ^3^Paul G. Allen School for Global Animal Health, Washington State University, Pullman, WA 99164, USA

## Abstract

Supervised machine learning algorithms are used by life scientists for a variety of objectives. Expert-curated public gene and protein databases are major resources for gathering data to train these algorithms. While these data resources are continuously updated, generally, these updates are not incorporated into published machine learning algorithms which thereby can become outdated soon after their introduction. In this paper, we propose a new model of operation for supervised machine learning algorithms that learn from genomic data. By defining these algorithms in a pipeline in which the training data gathering procedure and the learning process are automated, one can create a system that generates a classifier or predictor using information available from public resources. The proposed model is explained using three case studies on SignalP, MemLoci, and ApicoAP in which existing machine learning models are utilized in pipelines. Given that the vast majority of the procedures described for gathering training data can easily be automated, it is possible to transform valuable machine learning algorithms into self-evolving learners that benefit from the ever-changing data available for gene products and to develop new machine learning algorithms that are similarly capable.

## 1. Introduction

Public gene and protein databases such as GenBank [1], UniProt [2], and EuPathDB [3] are major resources for gathering data to train supervised machine learning algorithms used by life scientists for a variety of objectives including the detection of targeting sequences and the prediction of transmembrane domain topology. While these data resources are quite dynamic in nature, that is to say they are continuously updated by the addition of new information, machine learning applications are often static and cannot incorporate the new information. When a supervised machine learning approach is designed, by necessity the learning method is developed using the data available in public resources at the time of development, and it is then provided for public use. It is not uncommon to find algorithms in widespread use that were trained using data sets that were outdated soon after their introduction. Given that the vast majority of the procedures described for gathering training data can be easily automated—requiring very little, if any, human assistance—transforming machine learning algorithms into self-evolving learners that utilize new data on genes and proteins and developing new machine learning algorithms that are similarly capable is worthy of consideration.

In this study, we propose a new model of operation for specific supervised machine learning algorithms that is aligned with the fast changing nature of genomic data. We believe that applications that learn from genomic data should be defined in a pipeline in which the data gathering procedure for training data is automated and the learning process is as well. Such a pipeline would function as a classifier or predictor generator that does not require training data to be provided but instead is capable of generating a model from the information available from public resources at any given time.

Because every learning problem has its own training data requirements and data set curation procedures, the proposed model of operation is best illustrated using case studies. We selected three supervised machine learning methods and designed automated data gathering pipelines for each of them. Two of these pipelines are designed for the well-known SignalP 4.0 [4] and MemLoci [5] methods that are used to predict the location of signal peptide cleavage sites in amino acid sequences and the subcellular localization of eukaryotic membrane proteins, respectively. These two methods were selected due to their widespread use in the field and the size of the protein groups they apply to, which are quite large. For our third case study, in addition to designing the pipeline, we provided a full implementation which allowed us to discuss the design in more detail. For this case study, we selected the apicoplast-targeted protein prediction problem and utilized the existing machine learning model that we developed previously, ApicoAP [6], in a pipeline comprised of an automated training data gathering procedure and the classifier training routine defined as part of the ApicoAP model. Owning the source code for the ApicoAP training routine enabled us to provide the implementation of the pipeline. The client software for the ApicoAP pipeline can be found at http://bcb.eecs.wsu.edu/.

The apicoplast is a unique organelle found in a group of parasites known as Apicomplexa. Members of this parasite family include the causative agent of the most deadly form of malaria. The apicoplast is an essential organelle for the survival of these parasites, and due to its unique properties, its proteins are seen as promising drug targets [7, 8]. Therefore, identifying apicoplast proteins is important for the development of intervention therapies for diseases like malaria. ApicoAP predicts apicoplast proteins in multiple species of Apicomplexa, and with the development of ApicoAP Pipeline, its scope will increase to all the species whose genome sequences are available.

## 2. SignalP 4.0 Pipeline

SignalP 4.0 [4] predicts the presence and location of signal peptide cleavage sites in amino acid sequences of eukaryotes and both Gram-positive and Gram-negative prokaryotes. SignalP predictions are based on a combination of several artificial neural networks for which positive and negative training data are required to learn an accurate model of signal peptides for a class of organisms. Signal peptides are short sequences of amino acids that appear in proteins that are destined for a secretory pathway. Artificial neural networks are one of the most commonly used computational models capable of performing supervised machine learning tasks. As with many other machine learning methods, a training procedure is defined to learn from data.

**Figure 1:**
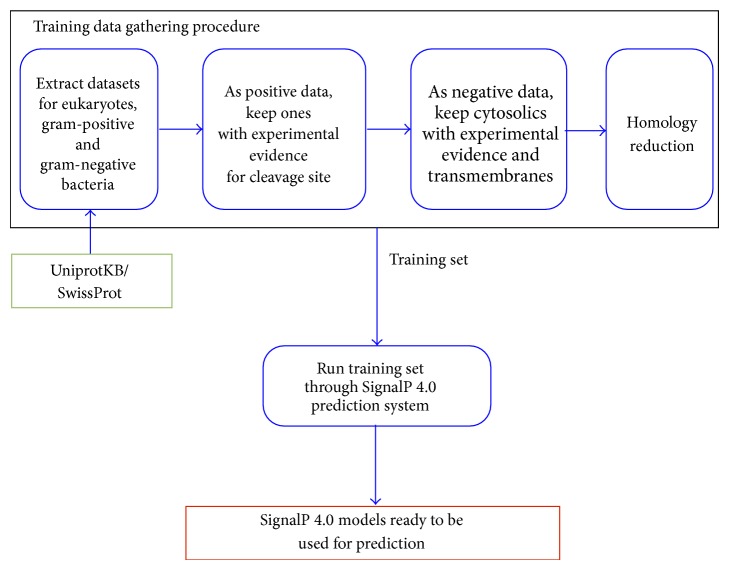
The SignalP 4.0 Pipeline.

The procedure used by SignalP 4.0 for data gathering can be fully automated and only depends on one external public resource, the UniprotKB/SwissProt database, which offers extensive web services for automated access. One can also download the latest release of the database and parse it through scripts. We designed a pipeline for SignalP 4.0 which includes automated training data gathering and model training operations.  Figure 1 shows a flowchart for the SignalP 4.0 pipeline. We provide a description of this flowchart below which should be viewed as a high-level summary. Details of the procedure can be found in the supplementary methods in [4].

SignalP requires three separate datasets with proteins of eukaryotes, Gram-positive bacteria, and Gram-negative bacteria. In every dataset, positive and negative subsets are defined through filters. The procedure for gathering training data begins with the extraction of protein sequences from UniprotKB/SwissProt. Restrictions are defined and enforced on the sequence length and inclusion of fragments. For positive subsets, sequences that have experimental evidence for cleavage sites are selected. This can easily be done by parsing protein entries in SwissProt that are meticulously annotated with such information.

For negative subsets, proteins with a subcellular location annotated as cytosolic with experimental evidence and transmembrane proteins with an annotated transmembrane region within the first 70 positions are selected. Selection can easily be done by parsing protein entries in SwissProt which have subcellular location and transmembrane status annotations in place.

After shortening all sequences to 70 N-terminal amino acids, homology reduction is carried out using algorithm 2 of [9] with custom cut-off criteria. The resulting training sets are used to train several artificial neural networks which yields SignalP 4.0 models ready to be used for prediction.

This pipeline can be maintained by setting up an update tracker on the UniprotKB/SwissProt database. Since a database version file can be downloaded via FTP, an update of the database can be detected using a simple script. Whenever the SwissProt database is updated, the data gathering procedure should be repeated. If the resulting training data are different than the previously created data, the model training routine should also be repeated to achieve the most up-to-date SignalP 4.0 model.

## 3. MemLoci Pipeline

MemLoci [5] predicts the subcellular location of membrane proteins into three membrane protein localization categories: plasma, internal, and organelle membrane. MemLoci is a support vector machine-based tool that requires training data for each prediction category to learn the discriminating hyperplanes between three classes. Support vector machines (SVM) are computational models commonly used to perform supervised machine learning tasks [10, 11]. The training procedure defined for SVMs produces a classifier by constructing an optimal hyperplane dividing the positive and negative classes with a maximum margin of separation. In the case of multiple classes, several SVM models are trained separating each class from the rest and a majority voting scheme is applied to reach the final prediction. SVMs have several hyperparameters that need to be optimized using separate testing data. Therefore MemLoci requires separate testing data in addition to training data.

**Figure 2:**
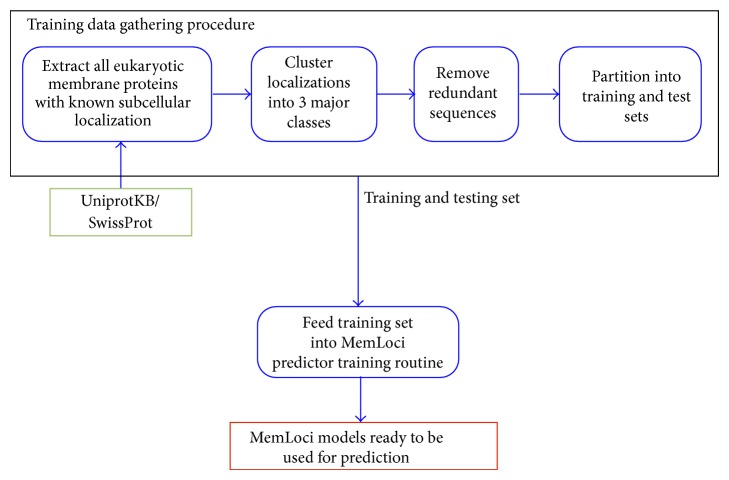
The MemLoci Pipeline.

As with SignalP 4.0, the procedure used by MemLoci for data gathering can be fully automated. It also depends on public resources, the UniprotKB/SwissProt database and BLAST [12]. The BLAST tool can be run locally by downloading its latest executable and databases. BLAST also offers extensive web services for automated access. We designed a pipeline for MemLoci which includes automated data gathering and model training operations.  Figure 2 shows a flowchart for the MemLoci pipeline. We provide a description of the pipeline below which is meant to be a high-level summary. Details of the procedure are given in [5].

The procedure for gathering data for MemLoci begins with the extraction of eukaryotic membrane proteins with known subcellular localization from UniprotKB/SwissProt by discarding sequences whose annotations are marked as PROBABLE, POSSIBLE, BY SIMILARITY, and FRAGMENT. Proteins from 10 different subcellular localizations are clustered into three localization categories according to a well-defined rule. Proteins that fall into more than one localization category are discarded.

In order to eliminate redundant sequences, a custom algorithm is carried out that requires all-against-all sequence alignments using BLAST [12]. The resulting set is partitioned into training and test sets. To insure test and training partitions share low similarity, the resulting dataset is clustered into subsets containing proteins with high sequence alignment scores with a transitive closure procedure. As proteins belonging to two different clusters share low similarity, 10 cross-validation sets are defined by randomly grouping these nonsimilar clusters. Cross-validation is carried out to determine the optimal hyperparameters to be used in SVM models. In each round of the cross-validation, 9 of the sets are used for training and one set is used for testing. During testing, prediction accuracy is calculated and average prediction accuracy over 10 rounds is used as the criterion to optimize. The optimal hyperparameter set is defined as the one that maximizes the optimization criterion. The parameters that have been determined are then used to conduct the final training which results in the MemLoci models used for prediction.

In addition to the previously explained maintenance routine regarding UniprotKB/SwissProt databases, the MemLoci pipeline requires an update tracker for BLAST databases. Any update to one of these databases should cause the data gathering procedure to be repeated. If the resulting training data are different than the previously created data, the model training routine should also be repeated to achieve the most up-to-date MemLoci model.

## 4. ApicoAP Pipeline

ApicoAP [6] is a generalized rule-based classification model that identifies apicoplast-targeted proteins that use a bipartite signaling mechanism (ApicoTPs). It uses a training set containing known ApicoTPs and non-ApicoTPs for a particular apicomplexan species as input, and its output is a classifier that identifies ApicoTPs from the proteins of this species. Predictions of SignalP 4.0 and MemLoci models are applicable to a class of proteins like eukaryotes, Gram-positive and Gram-negative bacteria. Unlike these, ApicoAP [6] models are trained with proteins of a specific apicomplexan species and known to work best over the proteins of this species.

The ApicoAP prediction software (version 2) currently provides service for 4 apicomplexan species but not for the remaining 13 apicomplexan species whose sequenced genomes are available at present. However, as ApicoAP is a generic model customizable to any apicomplexan species for which training data are available, it is possible to increase the number of species for which service is provided. In fact, if a procedure for gathering training data can be systematically defined and automated, one can utilize the ApicoAP model as part of a pipeline to employ proteome information for an apicomplexan species to create a classifier that identifies ApicoTPs from the proteins of this species. Moreover, as the results from experimental confirmation of ApicoTPs are published—the main resource for obtaining training data—such a pipeline will not only be useful for an apicomplexan species for which no ApicoAP predictor exists, but it will also provide ever-improving classifiers for apicomplexan species for which an ApicoAP predictor already exists.

In the remainder of this paper, we define a generic pipeline for the purpose of ApicoTP prediction (hereafter referred to as the ApicoAP pipeline) that consists of an automated training data gathering procedure and the ApicoTP classifier training routine. In addition, we discuss implementation of this pipeline, ApicoAP-CS for* Apico*AP Complete Suite, which is a collection of web services. ApicoAP-CS can be utilized to generate a species-specific ApicoAP classifier that can be easily integrated into the ApicoAP prediction software. ApicoAP-CS utilizes public databases such as ApiLoc [13], EuPathDB [3], and OrthoMCL [14] and public bioinformatics tools such as SignalP [15] and BLAST [12]. ApicoAP-CS client software is available at http://bcb.eecs.wsu.edu/ together with a video explaining its use.

**Figure 3:**
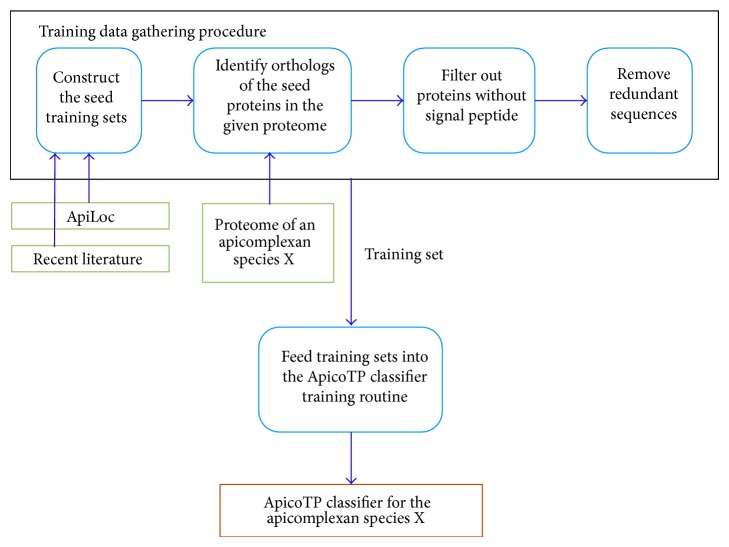
The ApicoAP Pipeline.

Figure 3 shows a flowchart for the ApicoAP pipeline. The procedure for gathering training data begins with the curation of two sets of proteins whose subcellular localization has been experimentally confirmed. These sets constitute the seed training sets and are used to identify the orthologs of the member proteins from the proteins of the apicomplexan species of interest. Known ApicoTPs and non-ApicoTPs for this apicomplexan species together with the orthologs of the seed training sets make up the interim training sets. A filtering step follows which eliminates proteins with no predicted signal peptide because the presence of a signal peptide is a requirement for ApicoTPs. The resulting sets are then screened for redundant sequences, which are removed, and the remaining elements form the final training sets. These sets are then fed into the ApicoTP classifier training routine which produces a species-specific ApicoTP classifier. Each of these steps is discussed in greater detail below.

### 4.1. Constructing Seed Training Sets

Seed training sets contain proteins whose subcellular localization has been experimentally confirmed. Because the ApicoAP model requires two training sets, one containing ApicoTPs (positive set) and the other non-ApicoTPs (negative set), two disjoint seed sets are needed. These sets may contain proteins from multiple apicomplexan species and they should be adequate to obtain sufficiently large final training sets. A discussion of the recommended sizes of the final training sets is given in Section 4.4.

The positive seed set consists of proteins that are known to localize to the apicoplast. The negative seed set consists of proteins that are known to localize to organelles in the cell other than the apicoplast. However, not all proteins confirmed to localize to nonapicoplast organelles are included in the latter set. For example, proteins confirmed to localize to the mitochondria, food vacuoles, the endoplasmic reticulum, and the cytoplasm are eliminated because dual localization incidents have been reported in the literature involving apicoplasts and these other locations.

ApiLoc [13] is an expert-curated database that currently serves as the main resource for curating apicomplexan proteins whose subcellular localization has been experimentally confirmed. ApicoAP-CS can automatically parse ApiLoc versions to extract the information necessary to prepare seed training sets. ApiLoc versions are provided to the public in the form of formatted spreadsheets. A web service interface to the database would make automated access to ApiLoc easier, but because such an interface is currently unavailable, ApicoAP-CS downloads the current version file using its URL and parses it via a Python script to retrieve protein records with identification and localization site information. Proteins localizing to the apicoplast as noted by ApiLoc are placed in the positive seed training set. Proteins localizing elsewhere as noted by ApiLoc, excluding the ones with dual localization incidents as mentioned previously, are placed in the negative seed training set. Because at present ApiLoc versions are not published very often, it may be beneficial to perform a literature search to insure that all possible information is present in the seed training sets. As such, ApicoAP-CS provides functionality for the user to submit additional proteins obtained from recent experimental studies. The user is urged to include only proteins whose localization has been experimentally confirmed. These proteins should also qualify to be either in the positive or the negative seed training set according to the criteria explained earlier.

### 4.2. Searching for Orthologs

Orthologs are defined as genes or gene products in different species which derive from a common ancestor [16]. Orthologs are expected to retain the same function in different species, thus having a strong likelihood of localizing to the same organelle in a cell. Therefore, utilizing orthology search strategies in training data gathering procedures, especially for subcellular localization prediction tasks, is a common practice.

Several approaches have been developed to predict putative orthologous proteins on the basis of different information sources including phylogenic relationships, protein-protein interaction networks, and sequence similarity relationships. While a simple BLAST (Basic Local Alignment Search Tool) search with a stringent *e*-value cut-off may identify a sequentially conserved subset of orthologs, other tools attempt to recognize orthology relationships in the event of low sequence conservation. Among orthologous protein prediction tools, OrthoMCL [14] focuses on eukaryotic genomes; it also has the most up-to-date support for apicomplexan species. EuPathDB [3] provides a user-friendly interface for orthology searches with OrthoMCL.

ApicoAP-CS utilizes the web service interface provided by EuPathDB to automatically identify orthologs of members of the seed training set in the proteome of an apicomplexan species. It provides an alternative ortholog search method for newly sequenced genomes that may not yet have EuPathDB or OrthoMCL support. This method uses a BLAST-based algorithm with a stringent *e*-value cutoff of 1*e* − 10; the best hits for each protein in the seed training sets are retained as orthologs. When possible, ApicoAP-CS uses both OrthoMCL and the BLAST-based algorithm to identify two separate ortholog sets and retains the union of these sets as the final ortholog set. The orthology search results in two interim training sets, the positive and negative training sets. At the conclusion of this step, proteins appearing in both sets are assumed to be caused by annotation errors and are discarded.

### 4.3. Filtering Proteins with No Signal Peptide

ApicoTPs must contain a signal peptide. To insure this requirement is met, we apply a filtering stage in which proteins not predicted to contain a signal peptide are discarded from both interim training sets. SignalP 3.0 is used to identify proteins with putative signal peptides because it is the tool commonly reported in the literature for apicomplexan genomes. ApicoAP-CS utilizes the web service interface of SignalP to automate the filtering step in the ApicoAP Pipeline. SignalP prediction results consist of a number of scores including* D*-Score and HMM probability, and a decision on whether a given sequence contains a signal peptide is made according to the default thresholds of the calculated scores. However, ApicoAP-CS does not use these default values. De Menezes Neto et al. [17] hypothesized that divergence in signal peptide predictions within orthologous groups is mainly due to N-terminal protein sequence misannotation and demonstrated that this is indeed the case. In addition to providing new gene models for certain proteins of* Plasmodium* spp, they suggested the use of thresholds differs from the default values for interpretation of SignalP [15]. We used their suggested threshold combination in ApicoAP-CS (*D*-Score = 0.48; HMM probability = 0.90). If one of these scores exceeds the corresponding threshold, the input protein is considered to contain a signal peptide. The Suds library, which provides a lightweight SOAP python client, was used for consuming the SignalP web service utility.

### 4.4. Removing Redundant Sequences

It is known that a training set containing redundant members biases the learning process [18, 19], especially when the learning involves an optimization procedure, as is the case for ApicoAP for which the optimization criterion is the expected performance of the candidate classifiers. The expected prediction performance of a classifier quantifies how well it is expected to generalize to new data instances. This performance metric can be estimated using statistical methods that involve retaining subsets of the training set from the training procedure and using these subsets to measure the prediction performance. These estimation strategies assume that the members of the training set are independently drawn from the main population. This assumption is violated with the existence of redundant members causing either overestimation or underestimation of the estimated performance.

ApicoAP-CS utilizes the CD-HIT (Cluster Database at High Identity with Tolerance) method (version 4.6.1) [20] to eliminate redundant sequences. CD-HIT takes a set of FASTA-formatted sequences as input and produces a set of representative sequences, having removed sequences that share an identity threshold (60% sequence similarity) with the retained sequences. CD-HIT avoids full alignment costs by applying a heuristic to find high identity segments between sequences. The resulting training sets with nonredundant sequences are subjected to user approval because expert knowledge may be required to identify unlikely ApicoTPs and non-ApicoTPs. ApicoAP-CS provides a user-friendly interface to review and edit the final training sets. If the final training sets have cardinality of about or exceeding 20, recommendation to proceed with the ApicoAP training is given; for lower cardinality, continuation to the next step is not recommended as the resulting classifier is not likely to have a high expected accuracy. As the cardinality and precision of the training sets increase, the expected accuracy of the resulting classifier improves.

### 4.5. Applying the ApicoTP Classifier Training Routine

The ApicoAP model describes a parametric model of ApicoTPs and utilizes an optimization/training procedure in which the parameters that will lead to the classifier with the best expected accuracy are identified. For a detailed description of ApicoAP, the reader is referred to [6]. ApicoAP-CS utilizes the same procedure, with the training sets obtained as described above, to generate an ApicoTP classifier specialized to the apicomplexan species of interest to the user. Once the ApicoTP classifier is generated, it can be used to identify putative ApicoTPs from the proteome of the given species. ApicoAP-CS provides the option to save the classifier information which can then be easily integrated into the ApicoAP prediction software described in [6]. A supplementary user's manual includes detailed instructions on the integration procedure for the ApicoAP prediction software (version 3). The training procedure takes considerably more time relative to the other steps in the ApicoAP Pipeline due to the computationally intensive optimization procedure required by the ApicoAP model.

### 4.6. External Dependencies of ApicoAP Pipeline and Maintenance

ApicoAP Pipeline depends on the following external public resources: ApiLoc, EupathDB, BLAST, and SignalP 3.0. In ApicoAP-CS, we run the BLAST tool locally. SignalP and EupathDB are accessed via web services they offer. Since ApiLoc does not provide web service interface, we download and parse the database without scripts.

In addition to the previously explained maintenance routine regarding BLAST databases, we plan to have a similar update tracker over ApiLoc database, as soon as the version information of this database is accessible via a standard web location. Version tracker for the EupathDB is also required for proper maintenance of the ApicoAP pipeline. Since SignalP 3.0 is a static methodology, there is no need to track updates on this tool.

## 5. ApicoAP-CS Results

We applied ApicoAP-CS to the 17 apicomplexan species whose genomes are available in EuPathDB (version 2.17), namely,* Babesia bovis*,* Babesia microti*,* Cryptosporidium hominis*,* Cryptosporidium muris*,* Cryptosporidium parvum*,* Eimeria tenella*,* Neospora caninum*,* Plasmodium berghei*,* Plasmodium chabaudi*,* Plasmodium cynomolgi*,* Plasmodium falciparum*,* Plasmodium knowlesi*,* Plasmodium vivax*,* Plasmodium yoelii*,* Theileria annulata*,* Theileria parva*, and* Toxoplasma gondii*. All protein sequences were obtained from EuPathDB (version 2.17) except the ones whose gene models were proposed to be changed in [17]. The positive seed training set contains 75 known ApicoTPs extracted from ApiLoc (version 3) and 18 confirmed proteins curated from recent literature. The current negative seed training set contains 400 known non-ApicoTPs extracted from ApiLoc.

We used both the OrthoMCL and BLAST-based algorithm (using Blast+ version 2.2.27) for the orthology search. Tables 1 and 2 show the cardinalities of positive and negative interim training sets that were automatically gathered by ApicoAP-CS. After the SignalP filtering step was applied, the resulting interim training sets (shown as the last column in Tables 1 and 2) were subjected to the redundancy removal procedure, which produced the final training sets whose cardinalities are shown in Table 3.

For 14 of the 17 apicomplexan species, we were able to gather sufficiently large training sets to train specialized ApicoTP classifiers. The remaining 3 apicomplexan species are of the* Cryptosporidium* genus, whose members are known to possess no apicoplast. These classifiers are included in the ApicoAP prediction software (version 3).  Table 4 gives the prediction accuracies for the resulting ApicoTP classifiers obtained using each training set. These are not expected accuracy results, which estimate how well the resulting classifiers will perform with unknown data, but rather they indicate how well these classifiers perform with the available, labeled data. Please note that the presented results are for illustration purpose only and are not meant to provide extensive information over the newly trained ApicoAP models. See supplementary Tables 1 and 2 available online at http://dx.doi.org/10.1155/2014/234236 (available from https://code.google.com/p/apicoapcs/downloads/detail?name=ApicoAP-CS_SupplementaryTables.xls&can=2&q=) for detailed information on the positive and negative seed training sets.

## 6. Discussion

Supervised machine learning algorithms that are provided for use by life scientists are often trained with data from expert-curated public gene and protein databases. While these data resources are continuously updated by the addition of new information, most machine learning algorithms are trained once using data sets that are outdated soon after their introduction.

In this paper, we proposed a new model of operation for specific supervised machine learning algorithms that utilize training sets curated from dynamically changing public resources, such as genomic databases. Given that the vast majority of the procedures described in the literature for gathering training data can easily be automated, it is possible to transform valuable machine learning algorithms into self-evolving learners that use ever-changing data on genes and proteins and to develop new machine learning algorithms that are similarly capable. We proposed a generic idea that involves employing these algorithms as part of a pipeline in which the training data gathering procedure and the training process are automated. By employing these algorithms as part of a pipeline, one can create a system that functions as a classifier generator that does not require the provision of training data but instead has the capability to utilize the information available from public resources for training. We demonstrated the practicality of this generic idea using three case studies for the well-known SignalP, MemLoci, and ApicoAP methods. Technically, the main requirement for automation of training data gathering is the ability to access the required genomic databases programmatically; this is provided by most public databases with web service interfaces. If these services are not updated any longer or change their APIs, affected pipeline designs would have to be updated. The promise of pipeline designs is not to provide software packages that will never become obsolete as this is an impossible goal. The practical promise here is to extend the life of a program, and such an extension may well be worth the implementation.

The SignalP and MemLoci Pipelines utilize the SignalP 4.0 and MemLoci methods and provide a high-level description of how to automate the training data gathering procedure. Current SignalP and MemLoci predictors are trained over datasets curated from 2010_05 and 2010_08 releases of UniProtKB/SwissProt, respectively. These predictors are still in common use while a 2014_07 release of UniProtKB/SwissProt is currently available. Implementations of these pipelines would offer up-to-date models for public use, and predictions would increase in accuracy with the addition of new information to the databases.

ApicoAP Pipeline utilizes the ApicoAP model [6] which provides a training procedure in which a species-specific classifier is generated using a training set that contains labeled proteins of the apicomplexan species of interest. The resulting ApicoAP classifier identifies apicoplast-targeted proteins of this species. The ApicoAP Pipeline is capable of generating classifiers for different apicomplexan species* without* requiring training data to be provided. As the results from experimental confirmation of apicoplast-targeted proteins are published—the main resource for obtaining training data—this pipeline will not only be useful for an apicomplexan species for which no ApicoAP classifier exists, but it will also provide ever-improving classifiers for apicomplexan species for which an ApicoAP classifier already exists.

The ApicoAP Pipeline was used to train ApicoAP classifiers for 10 more apicomplexan species in addition to the 4 existing ones that were provided by the ApicoAP prediction software. An implementation of this pipeline, ApicoAP-CS, is available as a collection of web services. ApicoAP-CS can be utilized to generate a species-specific ApicoAP classifier that can be easily integrated into the ApicoAP prediction software. ApicoAP-CS client software can be found at http://bcb.eecs.wsu.edu/.

## Supplementary Material

The Supplementary Material provides detailed information on the positive and negative seed training sets of proteins. Each protein is listed together with its description, identification number, organism, and the reason for including it in the training set.

## Figures and Tables

**Table 1:** Cardinalities of the positive interim training sets for the 17 apicomplexan species gathered by ApicoAP-CS.

Apicomplexan species	Ortho-MCL^a^	BLAST^b^	Confirmed^c^	All combined^d^	Conflicts removed^e^	Non-SP filtered^f^
*B. bovis *	46	45	4	61	59	18
*B. microti *	51	50	0	61	58	23
*C. hominis *	17	24	0	28	25	1
*C. muris *	19	27	0	32	29	0
*C. parvum *	17	24	0	29	26	2
*E. tenella *	82	61	1	89	84	30
*N. caninum *	78	68	0	82	77	21
*P. berghei *	72	73	0	77	73	49
*P. chabaudi *	72	73	0	77	72	51
*P. cynomolgi *	70	72	0	77	73	31
*P. falciparum *	45	60	40	89	85	52
*P. knowlesi *	72	72	0	77	73	49
*P. vivax *	69	72	0	75	71	51
*P. yoelii *	70	68	3	77	73	41
*T. annulata *	45	47	0	56	54	23
*T. parva *	49	42	0	59	57	25
*T. gondii *	53	59	45	102	96	42

^a^Cardinality of the set gathered by ortholog search using OrthoMCL.

^b^Cardinality of the set gathered by ortholog search using the BLAST-based algorithm.

^c^Cardinality of the set containing experimentally confirmed positive/negative proteins.

^d^Cardinality of the set that is the union of the sets presented in column 2, 3 and 4.

^e^Cardinality of the union set when conflicts with the negative/positive set is removed.

^f^Cardinality of the final training set after proteins without signal peptides have been removed.

**Table 2:** Cardinalities of the negative interim training sets for the 17 apicomplexan species gathered by ApicoAP-CS.

Apicomplexan Species	OrthoMCL^a^	BLAST^b^	Confirmed^c^	All Combined^d^	Conflicts Removed^e^	Non-SP Filtered^f^
*B. bovis *	144	136	8	161	159	33
*B. microti *	142	130	0	159	156	23
*C. hominis *	135	130	0	157	154	28
*C. muris *	143	137	0	163	160	34
*C. parvum *	130	129	10	164	161	33
*E. tenella *	400	175	8	443	438	169
*N. caninum *	254	220	15	288	283	81
*P. berghei *	222	212	28	260	256	101
*P. chabaudi *	238	223	2	258	253	108
*P. cynomolgi *	259	224	0	273	269	93
*P. falciparum *	284	173	156	443	439	138
*P. knowlesi *	236	227	6	258	254	91
*P. vivax *	261	227	13	281	277	103
*P. yoelii *	242	216	16	270	266	89
*T. annulata *	151	133	4	169	167	42
*T. parva *	186	128	4	204	202	71
*T. gondii *	194	198	131	333	327	92

^a^Cardinality of the set gathered by ortholog search using OrthoMCL.

^b^Cardinality of the set gathered by ortholog search using the BLAST-based algorithm.

^c^Cardinality of the set containing experimentally confirmed positive/negative proteins.

^d^Cardinality of the set that is the union of the sets presented in columns 2, 3, and 4.

^e^Cardinality of the union set when conflicts with the negative/positive set are removed.

^f^Cardinality of the final training set after proteins without signal peptides have been removed.

**Table 3:** Cardinalities of the final training sets for the 17 apicomplexan species.

Apicomplexan species	Positive training set	Negative training set
*B. bovis *	18	30
*B. microti *	23	22
*C. hominis *	1	28
*C. muris *	0	34
*C. parvum *	2	33
*E. tenella *	30	143
*N. caninum *	21	77
*P. berghei *	49	94
*P. chabaudi *	51	98
*P. cynomolgi *	31	90
*P. falciparum *	51	132
*P. knowlesi *	48	90
*P. vivax *	51	101
*P. yoelii *	41	87
*T. annulata *	23	41
*T. parva *	25	61
*T. gondii *	42	86

**Table 4:** ApicoTP classifier performances with the training sets gathered by ApicoAP-CS.

Apicomplexan species	True negative rate	True positive rate	Overall accuracy
* B. bovis *	1.000	1.000	1.000
*B. microti *	0.909	1.000	0.956
*E. tenella *	0.951	0.800	0.925
*N. caninum *	1.000	0.857	0.969
*P. berghei *	0.936	0.959	0.944
*P. chabaudi *	0.959	0.902	0.940
*P. cynomolgi *	1.000	0.839	0.959
*P. falciparum *	0.924	0.843	0.902
*P. knowlesi *	0.922	0.958	0.935
*P. vivax *	0.901	0.980	0.928
*P. yoelii *	0.954	0.854	0.922
*T. annulata *	0.854	0.913	0.875
*T. parva *	0.820	0.960	0.860
*T. gondii *	0.977	0.905	0.953
